# Microbiological Quality Assessment by PCR and Its Antibiotic Susceptibility in Mangrove Crabs (*Ucides cordatus*) from Guanabara Bay, Rio de Janeiro, Brazil

**DOI:** 10.1155/2016/7825031

**Published:** 2016-03-15

**Authors:** M. C. N. Carvalho, M. M. Jayme, G. S. Arenazio, F. V. Araújo, S. G. F. Leite, E. M. Del Aguila

**Affiliations:** ^1^Instituto de Química, Universidade Federal do Rio de Janeiro, Avenida Athos da Silveira 149, Bloco A, Cidade Universitária, 21949-909 Rio de Janeiro, RJ, Brazil; ^2^Faculdade de Ciências Médicas, Universidade do Estado do Rio do Janeiro (UERJ), Avenida Professor Manoel de Abreu 444, 2° andar, Vila Isabel, 20550-170 Rio de Janeiro, RJ, Brazil; ^3^Faculdade de Formação de Professores, Universidade do Estado do Rio de Janeiro (FFP-UERJ), Rua Dr. Francisco Portela 1470, Patronato, 24435-005 São Gonçalo, RJ, Brazil; ^4^Escola de Química, Universidade Federal do Rio de Janeiro (UFRJ), Avenida Athos da Silveira 149, Bloco E, Cidade Universitária, 21949-909 Rio de Janeiro, RJ, Brazil

## Abstract

The bacteriological quality of crabs from three different mangroves (Itaóca, Suruí, and Piedade) from Rio de Janeiro state, Brazil, was investigated using conventional and molecular methods. The results revealed high counts for total coliforms in meat and hepatopancreas samples. PCR analyses identified 25* Escherichia coli* colonies in the Itaóca, Piedade, and Suruí samples, detecting 13 enterotoxigenic colonies and 9 enteroaggregative colonies. Respectively, 12, 11, and 21* Vibrio parahaemolyticus* strains were detected in the Itaóca, Piedade, and Suruí samples. Two* V. cholerae* strains were detected in the Piedade samples. The* E. coli* strains isolated in the present study showed resistance to gentamicin.* E. coli* strains from the Piedade samples showed 33% resistance to chloramphenicol and the strains also showed multiresistance to several antimicrobial agents with a MAR index ranging from 0.12 to 0.31.* Vibrio* strains from Piedade, Itaóca, and Suruí showed 86%, 78%, and 85% resistance, respectively, to ampicillin. The isolated* Vibrio* strains showed multiresistance to several antimicrobial agents, with a MAR index ranging from 0.12 to 0.25. The presence of these organisms in crab meat is an indication of microbial contamination, which may pose health risks to consumers when improperly cooked.

## 1. Introduction

Brazil has ca. 8500 km of coastline with the second largest mangrove area on Earth [1]. These mangroves have suffered extensively with urbanization and industrialization in coastal regions, and, over the years, extensive ecosystems have disappeared, ending many of their important functions, such as being buffers against coastal erosion, retaining some pollutants, and being fishery areas [2].

Crabs are decapod crustaceans rich in sodium, potassium, and phosphorus with high amounts of iron, zinc, copper, and manganese. They also present high concentrations of vitamins A, C, B6, thiamine, and riboflavin and are considered a delicacy in several parts of the world [3]. Along the Brazilian coast, crabs are one of the most important natural resources in estuarine regions and can be intensely exploited without reaching an overfishing threshold, mainly because the picking method allows for the identification of the female individuals, which are of a different size compared to the males, and their release back into the environment [4].

Among the large and diverse range of mangrove products in the Brazilian north and north-eastern estuaries, the mangrove crab,* Ucides cordatus,* is the most harvested, with the highest commercial and subsistence importance to rural households of the coastal population [5]. Environment quality, as well as the mode of collection and processing of products, may affect the quantity and diversity of the microorganisms present on the surface of seafood and fishery products, which may cause increases in microbial contamination [6].

Beside the concern regarding the fecal contamination of human foods from marine ecosystems, starting in the late 1960s various indigenous bacteria from estuarine and marine waters were also recognized as potential human pathogens. They can be concentrated in shellfish, presenting human health risks [7]. The main concern is with regard to several species of* Vibrio*, such as* Vibrio parahaemolyticus*. Recent studies have also identified shellfish as sources of* Vibrio cholerae*,* Vibrio vulnificus*, and other* Vibrio* species in cases of human infections [8]. Some of these human pathogens can survive and grow at the low temperatures that characterize marine ecosystems.

*Vibrio*s are Gram-negative bacteria that are primarily associated with estuarine and coastal marine environments. A number of species have been associated with intestinal or extraintestinal infections in humans. All* Vibrio*s have an absolute requirement of Na^+^ for growth although some, such as* V. cholerae*, only require trace amounts. Only a small proportion of the* Vibrio*s belong to species potentially pathogenic in humans and, of these, only a small proportion may possess the pathogenicity traits that enable them to colonize and cause disease in the human body [9].

Marine* Vibrio*s naturally contaminating bivalve mollusks have been shown to be harder to remove by depuration than fecal bacterial indicators, such as* E. coli* [10]. Such processing methods may, therefore, not provide the necessary level of public health protection if significant levels of pathogenic* Vibrio*s are present in the harvested product.

*Escherichia coli* is a commensal microorganism whose niche is the mucous layer of the mammalian colon. It is the most abundant facultative anaerobe of the human intestinal microflora [11]. Furthermore,* E. coli* is widely distributed in the intestinal tracts of warm-blooded animals [12].* E. coli* is often nonpathogenic, although different strains may cause diseases in the gastrointestinal, urinary, or central nervous systems [13]. Currently, six categories of diarrheagenic* E. coli* have been acknowledged: enterotoxigenic* E. coli* (ETEC) [14], enteropathogenic* E. coli* (EPEC) [15], enteroinvasive* E. coli* (EIEC) [16], enterohemorrhagic* E. coli* (EHEC, Shiga toxin-producing* E. coli* or STEC) [17, 18], enteroaggregative* E*.* coli* (EAEC or EAggEc) [19], and diffusely adherent* E. coli* (DAEC) [20]. Despite not being very common, the isolation of diarrheagenic* E. coli* from seafood has been reported. In Brazil, Ayulo et al. (1994) [21] isolated only one strain of STEC from shellfish and gave evidence that preventive measures, especially during harvest and postharvest, are of major importance to avoid contamination of any nature.

Detection of pathogenic bacteria in seafood is essential to ensure safe products for consumers, sustainable fish, and shellfish growing activities. Molecular diagnostic methods have evolved significantly in the last few years and are now established as useful and reliable methods to allow the rapid detection and identification of pathogens. Molecular detection, identification, and enumeration of* Vibrio* spp. are largely based on PCR amplification following purification of nucleic acids from the samples. Although less sensitive and more time consuming, DNA or oligonucleotide probe-based hybridization methods have been proposed for the detection of* Vibrio* spp. in food [22].

Herein, the presence of potentially pathogenic isolates (*Vibrio* and* Escherichia coli* strains) from* Ucides cordatus* crabs from the Guanabara Bay, Rio de Janeiro, Brazil, is reported, using both conventional (biochemical identification) and molecular (PCR) methods. The antibiotic susceptibility of the isolates was also evaluated.

## 2. Material and Methods

### 2.1. Study Area

The mangroves selected for this study are located in Itaóca (São Gonçalo), Piedade (Mage), and Suruí (Mage), in Guanabara Bay, Rio de Janeiro, Brazil, where the gathering of this crustacean for marketing is more intense.

### 2.2. Sample Collection

Thirty live crabs (*Ucides cordatus*) were collected between March 2012 and June 2014 in each mangrove studied. These samples were analyzed at the Laboratory of Environmental Microbiology at the University of the State of Rio de Janeiro (UERJ). The crabs were washed to remove any excess sediment and other impurities present on their bodies. The viscera and meat were removed with a sterile forceps and a scalpel and placed into sterile Petri dishes. Twenty-five grams of each sample were mixed with 225 mL of buffered peptone water, and the suspensions were transferred to homogenizer bags (Interscience, Saint Nom, France) and coupled to a Stomacher® 400 circulator (Seward, Worthing, West Sussex, UK) at 260 rpm for 1 min [23]. The suspensions were serial-diluted from 10^−6^ to 10^0^ and 100 *μ*L of each dilution was transferred onto specific broths.

### 2.3. Microbiological Analyses of Crab Samples

The tests used for the determination of* E. coli* and* Vibrio* spp. are established in the Methods for the Microbiological Examination of Foods. The reference strains used as controls were provided by the Oswaldo Cruz Foundation, Rio de Janeiro, Brazil.

#### 2.3.1. Fecal Coliforms Analyses

Twenty-five grams of tissue were immersed in 225 mL of lactose broth (Himedia®, Mumbai, India) for 48 hours at 35°C. Subsequently, 10^−1^ to 10^−4^ dilutions were carried out with 9 mL of saline solution for posterior inoculation in lauryl sulfate broth (Himedia, Mumbai, India) at 35°C for 24 h. An 100 *μ*L aliquot of each positive tube lauryl sulfate broth (Himedia, Mumbai, India) was transferred to a corresponding tube containing 3 mL of EC broth (Himedia, Mumbai, India) with 5 Durham tubes for 24 hours with a series of dilutions and replicates in a water bath at 44.5°C [23] to determine the MPN (most probable number) coliform bacteria by counting.

#### 2.3.2. *Escherichia coli* Detection

An 100 *μ*L aliquot was removed from the tube containing 3 mL of positive EC broth (Merck®, Darmstadt, Germany) and transferred to agar plates containing EMB (Merck, Darmstadt, Germany). The plates were incubated for 24 hours at 37°C. The presumptive* E. coli* spp. colonies were submitted to biochemical tests: SIM (Sulfide-Indole-Motility) (BioBrás®, Minas Gerais, Brazil), citrate (Citrate of Simmons) (Difco®, Sparks, Maryland, USA), and MR/VP Broth (methyl red/Voges-Proskauer) (Merck, Darmstadt, Germany) [24].

#### 2.3.3. *Vibrio* spp. Detection

Twenty-five grams of crab meat and viscera were immersed in 225 mL of lactose broth (Himedia, Mumbai, India) for 48 hours at 35°C and transferred to 1 mL tubes containing BHI (Heart Brain Infusion) (Himedia, Mumbai, India) with 1% and 3% of NaCl and incubated for 24 h at 37°C. A 100 *μ*L aliquot was transferred to plates containing TCBS agar (Himedia, Mumbai, India) and were incubated for 24 h at 37°C. The presumptive* Vibrio* spp. colonies were submitted to biochemical characterization tests: oxidase test, Oxidation-Fermentation (OF) (Difco, Sparks, Maryland, USA), inositol (Difco, Sparks, Maryland, USA), and O129 (Celon-Lab®, Madhapur, Hyderabad, India) [25].

### 2.4. Molecular Analyses

#### 2.4.1. DNA Extraction

DNA preparation was carried out by the thermal shock method from all the harvested colonies. The colonies were grown in 3 mL of BHI broth harvested after 24 h at 37°C. One mL of the medium was transferred to sterile Eppendorf tubes and centrifuged for 10 min at 12,000 g. The supernatant was discarded and the pellet was resuspended in 400 *μ*L of pure sterile water. After homogenization, the supernatant was boiled for 10 min, cooled on ice for 5 min, and then collected and used for the PCR analyses [26].

#### 2.4.2. PCR Amplification for the* E. coli* Virulence Gene

PCR was performed using multiplex JMS1, LT, VirA, and EAE oligos and PCR-uniplex for AggRks and EAST1 oligos (Table 1). The reactions contained a final volume of 25 *μ*L containing 5 *μ*L of template DNA, buffer (10x), 10 mM dNTP, 25 mM MgCl_2_, 2 U Taq polymerase (Invitrogen Technologies®, São Paulo, Brazil), and 10 mM of each primer (Invitrogen Technologies, São Paulo, Brazil). The conditions of reaction were 94°C for 5 min, 30 cycles of 1 min at 94°C, 1 min at 58°C, 2 min at 72°C, and a final cycle of 72°C for 10 min, for all reactions. PCR amplicons were visualized on 2% agarose gels stained with 3 *μ*L of ethidium bromide (0.5 mg mL^−1^), visualized on a UV light transilluminator (Uvitec®, Cambridge, UK), and photodocumented by “Polaroid” (Canon®, São Paulo, Brazil).

#### 2.4.3. PCR Amplification for the* Vibrio* sp. Gene

The reaction was performed using multiplex oligos in a final volume of 20 *μ*L. The mixture contained 2 U Taq polymerase (Invitrogen Technologies, São Paulo, Brazil), 10 mM dNTPs, buffer (10x), 25 mM MgCl_2_, 3 *μ*L of template DNA, and 10 mM primers (sodB, sodB flaE, hsp, and 16S) (Table 2) [28]. The conditions of reaction were 5 min at 93°C followed by 35 cycles of 92°C for 40 s, 57°C for 1 min, and 72°C for 1.5 min and a final cycle at 72°C for 7 min, for all reactions. PCR amplicons were visualized on 2% agarose gels stained with 3 *μ*L of ethidium bromide (0.5 mg mL^−1^), visualized on a UV light transilluminator (Uvitec, Cambridge, UK), and photodocumented by “Polaroid” (Canon, São Paulo, Brazil).

### 2.5. Antibiotic Susceptibility Test

The microorganisms were inoculated at a concentration equivalent to 0.5 McFarland units (Probac®, Durban, South Africa) onto a Muller Hinton agar plate (Difco, Sparks, Maryland, USA). The antibiotic discs were placed on the plates and incubated overnight at 37°C. The inhibition zone was interpreted according to the Clinical Laboratory Standards M100-S22 Guidelines [29], formerly known as the National Committee for Clinical Laboratory Standards. The tested antibiotics were chloramphenicol (30 *μ*g), tetracycline (30 *μ*g), gentamicin (10 g), amikacin (30 *μ*g), tobramycin (10 g), trimethoprim-sulfamethoxazole (1.25/23.75 *μ*g), cephalothin (30 *μ*g), ampicillin (10 g), ceftazidime (30 *μ*g), cefotaxime (30 *μ*g), cefepime (30 *μ*g), aztreonam (30 *μ*g), cefoxitin (30 *μ*g), imipenem (10 g), ampicillin-sulbactam (10 *μ*g-10 *μ*g), and ciprofloxacin (5 g). For quality control,* E. coli* ATCC 25922 and* E. coli* ATCC 35218 were tested under the same conditions.

For strains confirmed as* Vibrio* spp., the test was performed according to the standard document M45-A2 [29], with the same antibiotic disks used for* E. coli* (Oxoid®, Hampshire, UK), with the exception of tobramycin (10 g) and aztreonam (30 *μ*g) and with the addition of levofloxacin (5 g) and ofloxacin (5 g).

The inhibition halos were measured with the aid of a millimeter ruler.

## 3. Results

### 3.1. Fecal Coliforms

High concentrations of fecal coliforms (6.2 × 10^2^ and 7.2 × 10^2^ NMP g^−1^) were found in the meat and hepatopancreas samples from the Itaoca mangrove, respectively. The samples from Piedade and Suruí mangroves showed concentrations of 2.4 × 10^2^ and 3.2 × 10^2^ NMP g^−1^ in meat samples, respectively, and 2.5 × 10^2^ and 3.5 × 10^2^ NMP g^−1^ in hepatopancreas samples, respectively. No significant difference was observed among the thermotolerant coliform values found in the meat and hepatopancreas samples between the mangroves (*p* < 0.05).

### 3.2. *Escherichia coli* Detection

**Figure 1:**
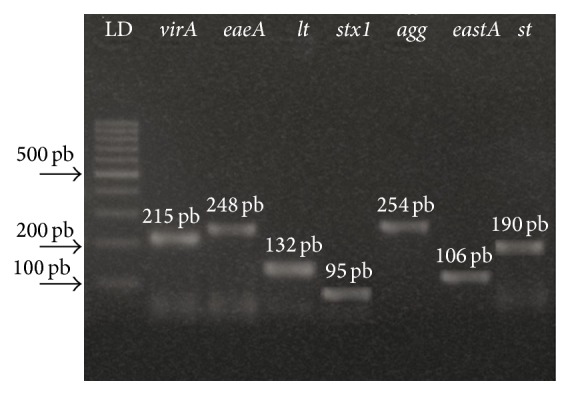
Specific amplicons of* E. coli* virulence genes.* E. coli* virulence gene (*virA*), enteroinvasive* E. coli* (*eaeA*), enteropathogenic* E. coli* (*lt* and* st*), enterotoxigenic* E. coli* (*stx1*), enterohemorrhagic* E. coli* (*astA*), and enteroaggregative* E. coli* (*agg*).

Multiplex PCR enabled the identification of 4 virulence genes (*eaeA*,* stx1*,* lt*, and* virA*) in single reaction (Figure 1).

Forty-six* E. coli* colonies isolated from the crab samples of the different mangroves (21 from meat and 25 colonies from hepatopancreas) were confirmed by biochemical tests. After biochemical characterization, the molecular test (PCR) revealed that 25 (54.3%) were positive for the researched virulence genes, 9 presenting* eastA* (36%), 13 presenting* lt* (52%), and 3 presenting* stx* (12%). No colonies presenting* virA*,* eaeA*,* st*, and* agg* genes were detected (Table 3).

Fourteen* E. coli* strains were isolated from Itaóca, with the presence of virulence genes, 2 presenting* stx1* (hepatopancreas), 7 presenting* lt* (4 in meat and 3 in hepatopancreas), and 5 presenting* eastA* (4 in meat and 1 in hepatopancreas). Eight strains were detected in samples from the Suruí mangrove, where 4 strains showed the* lt* virulence gene (2 in meat and 2 in hepatopancreas) and 4 strains showed the* east* virulence gene (2 in meat and 2 in hepatopancreas). Thirteen strains were detected in the samples from the Piedade mangrove by means of the biochemical test, but only one showed the presence of the* stx1* virulence gene (meat), while 2 showed the presence of the* lt* virulence gene (hepatopancreas).

### 3.3. *Vibrio* spp. Detection

**Figure 2:**
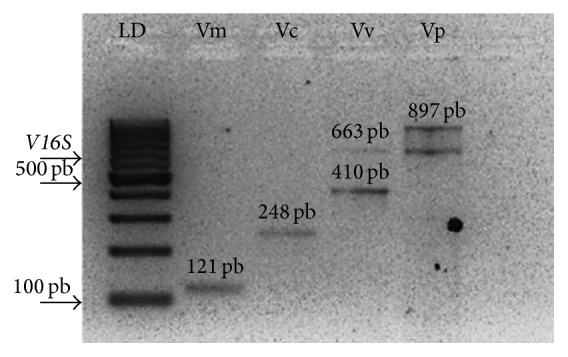
Amplicons of specific* Vibrio* genes. LD: molecular marker; Vm:* V. mimicus*, Vc:* V. cholerae*, Vv:* V. vulnificus*, and Vp:* V. parahaemolyticus.*

Suruí mangrove samples showed the highest incidence of isolated* Vibrio* (46), followed by Piedade (40) and Itaóca (33). One hundred and nineteen* Vibrio* strains were confirmed by PCR in 90 samples (meat: 68, and hepatopancreas: 51). The present study identified 5 different genes, one for the* Vibrio* spp. genus and 4 for species (Figure 2). A similar study was carried out by Teh et al. (2010) [30] using multiplex PCR (identifying the* gyrB* and* pntA* genes) to differentiate* V. parahaemolyticus*,* V. cholerae*,* V. vulnificus*, and other* Vibrio* spp. from fish.

Among the researched* Vibrio* genus, 61.3% (73/119) of the samples were detected using only the 16S gene for the Vibrionaceae family. Pathogenic strains* V. cholerae* and* V. parahaemolyticus* were found with a frequency of 1.7% (02/119) and 37% (44/119), respectively (Table 4).* V. cholerae* was only detected in the crab samples from Piedade mangrove. The highest incidence of* V. parahaemolyticus* was observed in samples from the Suruí mangrove (21), followed by Itaóca (12) and Piedade (11).

No* V. mimicus* and* V. vulnificus* were detected in the present study (Table 4).

### 3.4. Antimicrobial Susceptibility Test

The resistance results are displayed in Table 5. Twenty-six* E. coli* strains showed some resistance to the tested antimicrobials, with a high index of resistance.* E. coli* strains isolated from the Itaóca samples showed high resistance (63%) against gentamicin (CN) and tobramycin (TOB).

*E. coli* strains found at Piedade, Itaóca, and Suruí showed resistance to gentamicin (66%, 63%, and 22%, resp.). The* E. coli* isolates from the Piedade samples showed 33% resistance to chloramphenicol (C). Only strains found in crabs from the Piedade mangrove showed resistance (16%) to ampicillin (AMP). No resistance to amoxicillin + clavulanic acid (AMC), levofloxacin (LEV), cefoxitin (CTX), ofloxacin (OFX), and ciprofloxacin (CIP) was observed.

Among the 26 resistant* E. coli* strains, 12 were resistant to two or more antibiotics (Table 6). This pattern is mainly due to the indiscriminate use of antimicrobials and may cause serious impacts on human health [31, 32]. The* E. coli* strains showed multiresistance to several antimicrobial agents, with MAR indices ranging from 0.12 to 0.31, whereas 3 strains showed MAR indexes from 0.12 to 0.25 and 3 strains presented MAR indexes of 0.18 (Table 6). The resistance of the 26* E. coli* strains was distributed as follows: 12 strains were resistant to gentamicin and tobramycin, 4 were resistant to amikacin and cephalothin, 3 were resistant to ciprofloxacin, tetracycline, ceftazidime, and cefoxitin, and one strain was resistant to ampicillin.

When 119* Vibrio* strains were analyzed only 72 isolates (60.5%) showed resistance to some of the tested antimicrobials, with higher rates in those isolated from crabs samples from Piedade (29), followed by Itaóca (23) and Surui (20) (Table 5). The* Vibrio* strains from Piedade, Itaóca, and Suruí showed resistance to ampicillin (86%, 78%, and 85%, resp.). The strains from Surui showed 5% resistance to amoxicillin + clavulanic acid (AMC), ampicillin/sulbactam (SAM), and chloramphenicol (C). The Piedade strains showed resistance to levofloxacin (LEV) and ciprofloxacin (CIP). No resistance to cefoxitin (CTX), ceftazidime (CAZ), tobramycin (TOB), and tetracycline (TE) was observed.

*Vibrio* strains isolated from crabs showed multiresistance to several antimicrobial agents, presenting a MAR index ranging from 0.12 to 0.25; 24 strains presented MAR indices of 0.12 (Table 6); 5 strains showed MAR indices of 0.18; and two strains showed MAR indices of 0.25 MAR indexes.

The resistance of the 72 strains was distributed as follows: 60 strains were resistant to ampicillin, 14 were resistant to amikacin (AK), 10 were resistant to cephalothin (KF), 8 were resistant to cefoxitin (CTX), 5 were resistant to gentamicin (CN), 3 were resistant to ciprofloxacin (CIP), and 1 strain was resistant to amoxicillin + clavulanic (AMC), ampicillin + sulbactam (SAM), levofloxacin (LEV), ofloxacin (OFX), and chloramphenicol (C).

## 4. Discussion

The thermotolerant coliforms found in the present study are above the maximum permissible limit (maximum tolerance of 5 × 10^1^ NMP g^−1^ for coliforms at 45°C) in bivalve mollusks, crab meat, and similar samples, according to laws from the Brazilian Sanitary Vigilance Agency (Agência Nacional de Vigilância Sanitária (ANVISA)) [33]. Similar results were found with regard to the microbiological quality of Úça crab meat in 3 different points at Praia do Futuro, located in Fortaleza, CE, Brazil, where thermotolerant coliforms were detected ranging from 3.0 to 1,100 NMP g^−1^ in 90 analyzed crabs [34]. According to de Lima Grisi and Gorlach-Lira (2010) [35], the presence of this group of bacteria is associated to the dumping of fecal material in the environment. Guanabara Bay receives effluents without treatment daily and has become bacteria reservoir, which in turn has caused the contamination of fish and other biota in this region [36].

The expression of* E. coli* virulence genes is a public health risk, since these genes characterize the presence of toxins able to cause disease.* E. coli* cells are the main pathogens associated to gastroenteritis of food origin in humans, provoking diarrhea, hemorrhagic colitis, and hemolytic-uremic syndrome [37]. However, some studies reporting human infection by* E. coli* due to crab consumption are available [38]. Despite the absence of the* virA*,* eae*,* st*, and* agg* genes in the present study, the confirmation of* E. coli* strains indicates recent fecal contamination in crabs, and this indicates that major care in the preparation of this type of food is required.

The results regarding the presence of* Vibrio* can be explained by the salinity and temperature of the studied mangroves [39]. Many studies show the presence of* Vibrio* in aquatic animals such as fish [40], shrimp [41], and mussels [42], but, despite the importance of crabs, only some studies have been conducted on crab contamination by* Vibrio*. However, its occurrence in marine food is pointed as a major cause of gastroenteritis in the United States and Europe [43] and associated with cases in Brazil and Chile [44]. These results suggest a probable health risk for people that consume raw and undercooked seafood. According to Alam et al. (2012) [45],* V. vulnificus* and* V. mimicus* are most commonly found in coccoid viable but not culturable form, while another study confirmed the presence of* Vibrio* in crabs marketed in Fortaleza, Brazil, where only 10 strains were identified up to the species level: 2* V. alginolyticus* and 8* V. parahaemolyticus* but not any* V. vulnificus* and* V. mimicus* [4]. Abd-Elghany and Sallam (2013) [46] detected 10* V. parahaemolyticus* isolates in crab by molecular identification in Egypt and highlighted that reliable molecular detection methods should be included in routine seafood examinations, in addition to the conventional bacteriological methods.

These findings of antimicrobial susceptibility are in agreement with data from previous studies, which found that resistance to aminoglycosides, *β*-lactamase, and penicillin is common among* E. coli* isolates from food of animal origin [47–49]. However, the resistance frequency in* E. coli* isolated in the present study was low when compared to other studies, where a resistance of 58% and 42% in raw fish samples from Kenya and Vietnam, respectively, was observed [48, 50]. Mussel samples from Niterói (Brazilian Southeastern oceanic region) showed 29% resistance to at least one antimicrobial [31], and strains isolated from mussels from the Guanabara Bay, Rio de Janeiro, showed 40% to 85% resistance to tested antimicrobials [51], indicating the intense presence of domestic and industrial effluents. The percentage of high sensitivity to these antibiotics was also observed by Rebouças et al. (2011) [41] in strains isolated from shellfish and is associated with various resistance mechanisms found in Gram-negative organisms. Over time,* Vibrio* strains exposed to antibiotics through the environment can acquire antimicrobial resistance transferable by mobile genetic elements and horizontal gene transfer [52]. Thus, due to the presence of R-factors in the population, resistance developed through gene regulation of plasmids and chromosomes may be transferred vertically (by heredity) or horizontally [53]. In the present study, ampicillin was an antibiotic that showed low efficiency against the 60 tested* Vibrio* spp. strains (83.3%). According to the standard CLSI M45-A2 [54], species belonging to the* Vibrio* genus have intrinsic resistance to ampicillin. This data was confirmed in another study, where, from 169* Vibrio* strains isolated from shrimp, only 3 were sensitive to ampicillin [55]. The high percentage of pathogenic* Vibrio* with reduced susceptibility to ampicillin suggests a potential for the low efficiency of ampicillin in the treatment of* Vibrio* infections [56].

Many cases of multiple antimicrobial resistance have been reported from shellfish farms in countries where the activity is well developed, such as China [57], Korea [58], and Chile [59]. According to the World Health Organization, changes in the microbiota can induce the evolution of new pathogenic microorganisms and the development of new virulence factors in ancient pathogens, such as the development of resistance to antimicrobials or changes in their survival ability in adverse environmental conditions [60].

## 5. Conclusions

Several* E. coli* and* Vibrio* isolates were found in crabs (*Ucides cordatus*) from different mangroves in the state of Rio de Janeiro, Brazil. Considering the current legislation, the presence of these pathogens in crab indicates contamination influenced by mangrove pollution, by using newer molecular methods and thus contributing to seafood safety. Some isolated strains showed differential resistance to antimicrobials. The analyzed samples presented unsuitable hygienic-sanitary conditions, which can be considered a warning to the Municipal Health Surveillance Agency, since seafood is many times consumed without any subsequent thermal treatment or even sufficient thermal treatment able to eliminate pathogenic microorganisms, causing disorders to consumer health.

## Figures and Tables

**Table 1:** Primer sequences used for the identification of *Escherichia coli* virulence.

Serotypes	Oligonucleotides	Gene	Sequences (5′-3′)	Fragment size (bp)
*EPEC*	EAE-a	*eaeA*	ATG CTT AGT GCT GGT TTA GG	248
	EAE-b		GCC TTC ATC ATT TCG CTT TC	

*EHEC*	JMS1-F	*stx1*	GTC ACA GTA ACA AAC CGT AAC A	95
	JMS1-R		TCG TTG ACT ACT TCT TAT CTG GA	

*ETEC*	LT-1	*lt*	AGC AGG TTT CCC ACC GGA TCA CCA	132
	LT1-2		GTG CTC AGA TTC TGG GTC TC	
	Sta-F	*st*	GCT AAT GTT GGC AAT TTT TAT TTC TGT A	190
	Sta-R		AGG ATT ACA ACA AAG TTC ACA GCA GTA A	

*EAEC*	Aggrks-1	*aggR*	GTA TAC ACA AAA GAA GGA AGC	254
	Aggrks-2		ACA GAA TCG TCA GCA TCA GC	
	East1s	*astA*	GAG TGA CGG CTT TGT AGT CC	106
	East1sa		GCC ATC AAC ACA GTA TAT CC	

*EIEC*	VirA-F	*virA*	CTG CAT TCT GGC AAT CTC TTC ACA	215
	VirA-R		TGA TGA GCT AAC TTC GTA AGC CCT CC	

The pathotypes and virulence genes for the *E. coli* detected in this study are EPEC: enteropathogenic *E. coli*, EHEC: enterohemorrhagic *E. coli*, ETEC: enterotoxigenic *E. coli*, EAEC: enteroaggregative *E. coli*, and EIEC: enteroinvasive *E. coli* (adapted from Bisi Johnson et al. 2011 [27]).

**Table 2:** Oligonucleotide sequences used for the identification of the *Vibrio* genus and serotypes.

Serotypes	Oligonucleotides	Gene	Sequences (5′-3′)	Fragment size (bp)
*Vibrio *spp.	V.16S-700F	*16S*	CGG TGA AAT GCG TAG AGA T	663
	V.16S1325R		TTA CTA GCG ATT CCG AGT TC	

*V. cholerae*	Vc.sodB-F	*sodB*	AAG ACC TCA ACT GGC GGT A	248
	Vc.sodB-R		GAA GTG TTA GTG ATC GCC AGA GT	

*V. mimicus*	Vm.sodB-F	*sodB*	CAT TCG GTT CTT TCG CTG AT	121
	Vm.sodB-R2		GAA GTG TTA GTG ATT GCT AGA GAT	

*V. parahaemolyticus*	Vp.flaE-79F	*flaE*	GCA GCT GAT CAA AAC GTT GAG T	897
	Vp.flae-934R		ATT ATC GAT CGT GCC ACT CAC	

*V. vulnificus*	Vv.hsp-326F	*hsp*	GTC TTA AAG CGG TTG CTG C	410
	Vv.hsp-697R		CGC TTC AAG TGC TGG TAG AAG	

**Table 3:** Expression of *E. coli *virulence genes by PCR distributed by mangrove.

Strains	Gene	Itaóca (*n* = 14)	Piedade (*n* = 3)	Suruí (*n* = 8)
Enteropathogenic	*eaeA*	0	0	0
Enterohemorrhagic	*stx1*	2	1	0
Enterotoxigenic	*lt*	7	2	4
	*st*	0	0	0
Enteroaggregative	*agg*	0	0	0
	*eastA*	5	0	4
Enteroinvasive	*virA*	0	0	0

**Table 4:** Distribution of *Vibrio* strains by mangrove.

Strains	Gene	Mangrove			
		Itaóca (*n* = 33)	Piedade (*n* = 40)	Suruí (*n* = 46)	Total (*n* = 119)
*Vibrio *spp.	*16S*	21	27	25	73
*V. cholerae*	*sodB*	0	02	0	02
*V. parahaemolyticus*	*flaE*	12	11	21	44
*V. mimicus*	*sodB1*	0	0	0	0
*V. vulnificus*	*hsp*	0	0	0	0

*n*: number of strains.

**Table 5:** * E. coli* and *Vibrio *resistance of strains isolated from crab to the tested antimicrobials.

Antimicrobial agent resistance	*E. coli*			*Vibrio*		
	Itaóca (*n* = 11)	Piedade (*n* = 06)	Suruí (*n* = 09)	Itaóca (*n* = 23)	Piedade (*n* = 29)	Suruí (*n* = 20)
AMP	—	16%	—	78%	86%	85%
AMC	—	—	—	—	—	5%
SAM	—	16%	11%	—	—	5%
KF	19%	16%	11%	8%	17%	15%
CTX	—	—	—	13%	17%	—
CN	63%	66%	22%	4%	6%	10%
CFO	9%	16%	—	—	—	—
CAZ	9%	16%	—	—	—	—
LEV	—	—	—	—	3%	—
OFX	—	—	—	4%	—	—
TOB	63%	66%	22%	—	—	—
AK	—	33%	22%	17%	24%	15%
TE	—	16%	11%	—	—	—
CIP	—	—	—	—	3%	10%
C	9%	33%	—	—	—	5%

*n*: number of tested strains. AMP: ampicillin; AMC: amoxicillin + clavulanic acid; SAM: ampicillin/sulbactam; KF: cephalothin; CTX: cefotaxime; CFO: cefoxitin; CAZ: ceftazidime; LEV: levofloxacin; CIP: ciprofloxacin; OFX: ofloxacin; CN: gentamicin; TOB: tobramycin; AK: amikacin; TE: tetracycline; C: chloramphenicol.

**Table 6:** Multiple antimicrobial resistance of *E. coli* and *Vibrio* strains found in crab.

	Antimicrobial resistance	MAR index
*E. coli*		
(3)	CN, TOB	0.12
(1)	CFO, TOB	0.12
(1)	CN, KF, TOB	0.18
(2)	AK, CN, TOB	0.18
(1)	C, CAZ, CN, TOB	0.25
(1)	C, CN, KF, SAM	0.25
(1)	CN, CFO, KF, TOB	0.25
(1)	AK, AMP, C, CAZ, CN, TOB	0.31
(1)	AK, CN, KF, SAM, TE, TOB	0.31
*Vibrio*		
(14)	AK, AMP	0.12
(6)	AMP, KF	0.12
(2)	AK, KF	0.12
(2)	CTX, KF	0.12
(2)	AK, CN, CTX	0.18
(1)	AK, CTX, KF	0.18
(2)	CN, CTX, KF	0.18
(1)	AK, CIP, KF, LEV	0.25
(1)	AK, CTX, CN, KF	0.25

The MAR (multiple antimicrobial resistance) index of an isolate is defined as *a*/*b*, where *a* represents the number of antibiotics to which the isolate was resistant and *b* represents the number of antibiotics to which the isolate was subjected. AK: amikacin; AMP: ampicillin; AMC: amoxicillin + clavulanic acid; C: chloramphenicol, CAZ: ceftazidime; CFO: cefoxitin; CIP: ciprofloxacin; CN: gentamicin; CTX: cefoxitin; KF: cephalothin; LEV: levofloxacin; OFX: ofloxacin; SAM: ampicillin/sulbactam; TOB: tobramycin; TE: tetracycline.
